# Methodology for Quantitative Characterization of Fluorophore Photoswitching to Predict Superresolution Microscopy Image Quality

**DOI:** 10.1038/srep29687

**Published:** 2016-07-14

**Authors:** Amy M. Bittel, Andrew Nickerson, Isaac S. Saldivar, Nick J. Dolman, Xiaolin Nan, Summer L. Gibbs

**Affiliations:** 1 Biomedical Engineering Department, Oregon Health & Science University, Portland, OR 97201, USA; 2 Thermo Fisher Scientific, Eugene, OR 97402, USA; 3 Knight Cancer Institute, Oregon Health & Science University, Portland, OR 97201, USA; 4 OHSU Center for Spatial Systems Biomedicine, Oregon Health & Science University, Portland, OR 97201, USA.

## Abstract

Single-molecule localization microscopy (SMLM) image quality and resolution strongly depend on the photoswitching properties of fluorophores used for sample labeling. Development of fluorophores with optimized photoswitching will considerably improve SMLM spatial and spectral resolution. Currently, evaluating fluorophore photoswitching requires protein-conjugation before assessment mandating specific fluorophore functionality, which is a major hurdle for systematic characterization. Herein, we validated polyvinyl alcohol (PVA) as a single-molecule environment to efficiently quantify the photoswitching properties of fluorophores and identified photoswitching properties predictive of quality SMLM images. We demonstrated that the same fluorophore photoswitching properties measured in PVA films and using antibody adsorption, a protein-conjugation environment analogous to labeled cells, were significantly correlated to microtubule width and continuity, surrogate measures of SMLM image quality. Defining PVA as a fluorophore photoswitching screening platform will facilitate SMLM fluorophore development and optimal image buffer assessment through facile and accurate photoswitching property characterization, which translates to SMLM fluorophore imaging performance.

Superresolution microscopy (SRM) has enabled fluorescence imaging at unprecedented spatial resolution^1^,^2^,^3^. Although a number of SRM techniques exist, single-molecule localization microscopy (SMLM) has gained in popularity due to its relative ease of instrumentation and compatibility with current labeling approaches for biological samples^4^,^5^. Two common SMLM techniques are stochastic optical reconstruction microscopy (STORM)^2^,^6^,^7^ and photoactivated localization microscopy (PALM)^1^,^8^, which enable visualization with ~10–20 nm resolution; however, resolution depends strongly upon the performance of the fluorophore used for sample labeling^9^,^10^,^11^,^12^.

SMLM requires dense labeling of features of interest with photoswitchable fluorophores that have the ability to stochastically switch between the fluorescent “on” state where photons are emitted and the nonfluorescent “off” or dark state^13^,^14^. Although the exact photoswitching mechanism is unknown for all fluorophore scaffolds, switching between the fluorescent on and off states is largely attributed to manipulation of the reductive and oxidative status of the imaging environment^13^,^15^,^16^. Subdiffractive localization of individual fluorophores throughout a series of images via activation of a stochastic, small population of fluorophores in the on state facilitates reconstruction of a superresolution image^17^. Thus, the photoswitching ability of the fluorescent labels is crucial to the quality of SMLM.

Currently utilized fluorophores largely consist of commercially available probes that have been demonstrated to photoswitch or be photoactivatable under certain imaging conditions^10^,^18^,^19^. However such probes are not designed to provide the optimal photoswitching as it is a disadvantage for conventional fluorescence microscopy, presenting an opportunity for photoswitching improvement. The most widely used fluorophores for SMLM include Alexa Fluor 647 and Cy5, which can achieve ~20 nm resolution, yet the majority of commercial fluorophores identified as the top SMLM candidates reach resolutions of only 30–40 nm at best^6^,^10^,^18^. Furthermore, as SMLM progresses to imaging multi-protein cellular complexes with multicolor SMLM, photoswitchable fluorophores with relatively narrow excitation and emission spectra will be required to minimize crosstalk^10^,^18^. Advancing the spatial and spectral resolution of SMLM will be realized through the synthesis of fluorophores specifically designed to photoswitch or photoactivate with ideal spectral properties for multicolor imaging. The chemical space to be investigated for development of ideal photoswitchable fluorophores in addition to the numerous imaging buffer formulations that significantly impact photoswitching behavior present a pressing need for a robust and efficient method to characterize fluorophore utility for SMLM.

A common approach to assess fluorophore photoswitching is through *in vitro* labeling of a known cellular structure in cells with descriptive image evaluation of the rendered SMLM image as this closely reflects most SMLM fluorophore applications^10^,^18^,^20^. However, the quality of the resulting image is often influenced by more than just fluorophore photoswitching properties. SMLM image quality can also reflect labeling issues arising from nonspecific binding or insufficient labeling density rather than the photoswitching ability of the fluorophore. Descriptive image evaluation of SMLM image quality is subjective and thus not readily comparable between fluorophores. Additionally, *in vitro* evaluation requires the fluorophore of interest to contain a readily conjugatable group, such as an N-hydroxysuccinimide (NHS) ester, maleimide or azide, for conjugation to a targeting molecule where the type of chemical attachment to the labeling protein may affect fluorophore photoswitching. Thus, *in vitro* evaluation of photoswitching requires additional fluorophore synthetic steps for conjugation, convolves the fluorophore photoswitching properties with labeling density and nonspecific background, and may be affected by the selected protein attachment strategy, making direct assessment of fluorophore photoswitching time consuming and largely descriptive instead of quantitative.

An alternative approach is studying fluorophore photophysical properties sans cells by spatially isolating and immobilizing single molecules. Such single-molecule systems include fluorophore adsorption to coverglass through protein-conjugation^18^,^21^,^22^,^23^,^24^ and fluorophores fixed in various polymer films^25^,^26^,^27^,^28^,^29^,^30^,^31^. Protein adsorption may more closely represent the environment utilized for biological SMLM imaging than polymer films while minimizing sample preparation time in comparison to evaluation of photoswitching properties in cells. However, protein adsorption still requires addition of a readily conjugatable group on the fluorophore of interest, where the chosen chemical conjugation strategy may affect photoswitching properties. By contrast, polymer films enable isolation of fluorophores devoid of conjugatable groups, which has significant utility for rapid screening of novel fluorophores as a photoswitch as well as isolation of the photoswitching properties from conjugation strategy. While fluorophore photoswitching performance evaluated via protein adsorption has been shown to qualitatively correlate to cell imaging applications^18^, it has not been established how the more advantageous polymer film isolation method compares, nor how photoswitching properties quantitatively compare to SMLM image quality.

In this study, we compared the polymer film and the protein adsorption single-molecule systems for their ability to predict *in vitro* SMLM image quality within cells through measurement of photoswitching properties. SMLM images ranging in quality were acquired of microtubules labeled in cells with eight commercial fluorophores. Photoswitching properties of the eight fluorophores were then obtained using dilute fluorophores dried into a polyvinyl alcohol (PVA) film and fluorophore-conjugated antibodies adsorbed to coverglass as the two single-molecule isolation platforms. Through statistical analysis, we identified the photoswitching properties measured using each isolation platform that correlate to, and ultimately predict, SMLM image quality. We demonstrated PVA films were efficacious and robust in evaluating fluorophores for SMLM imaging applications and hence, provide the necessary screening system for SMLM fluorophore development.

## Results

### SMLM Image Quality Varies with Photoswitchable Fluorophore Label

We established *in vitro* SMLM performance of selected fluorophores through quantitative assessment of the structure of microtubules labeled with indirect immunofluorescence in fixed cells. Eight commercially available fluorophores were used: three blue absorbing fluorophores, Fluorescein, ATTO 488 (ATTO488), and BODIPY™ FL (BODIPY FL); two green absorbing fluorophores, Cy™3B (Cy3B), and Alexa Fluor™ 568 (AlexaFluor568); and three red absorbing fluorophores, Cy™5 (Cy5), Alexa Fluor™ 647 (AlexaFluor647), and ATTO 680 (ATTO680). These eight fluorophores covered the ultraviolet to red region of the light spectrum and represented four classic fluorophore scaffolds including xanthene, BODIPY, cyanine, and oxazine. Microtubule labeling was completed with equal concentrations of primary antibody and fluorophore concentration conjugated to secondary antibody to maintain equal labeling density across the different fluorophores. Images of all fluorophores were collected under constant imaging buffer, image capture settings, and fluence rate within an excitation laser line.

**Figure 1:**
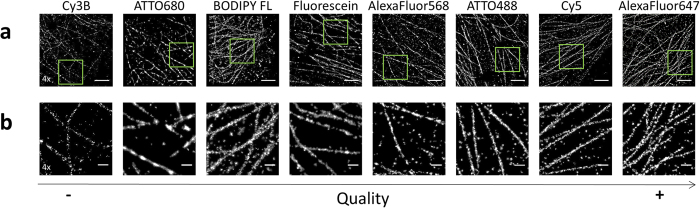
SMLM imaging of microtubules *in vitro* via indirect immunofluorescence in fixed cells. (**a**) Rendered SMLM images of microtubules (scale bar = 2 μm) with (**b**) magnified boxes to demonstrate reconstructed microtubule detail (scale bar = 0.5 μm). Panels labeled with “4x” are displayed with four times the brightness in comparison to other panels. Rendered images were organized by increasing observed quality from left to right: Cy3B, ATTO680, BODIPY FL, Fluorescein, AlexaFluor568, ATTO488, Cy5, and AlexaFluor647.

**Figure 2:**
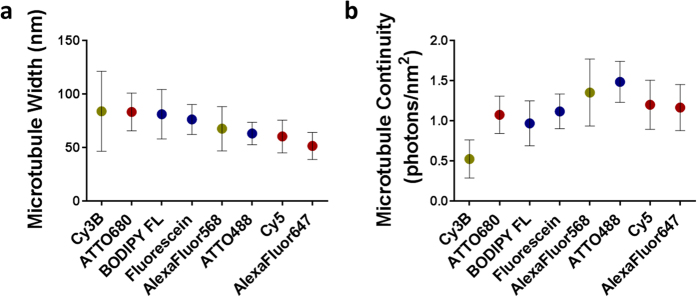
Calculated (**a**) average microtubule width and (**b**) average continuity of microtubules for each fluorophore, organized by observed quality. Average width and continuity measurements are graphed as the mean ± standard deviation of n = 15 measurements per image of the corresponding fluorophore. Images were collected at the following fluence rates: 0.28 kWcm^−2^ for the 488 nm laser line (blue points), 0.49 kWcm^−2^ for the 561 nm laser line (green points) and 1.11 kWcm^−2^ for the 647 nm laser line (red points).

Microtubule structures were visible in all rendered images with image quality varying in fluorophore homogeneity (Fig. 1), measured width (Fig. 2a), and measured continuity (Fig. 2b) of the labeled structure. AlexaFluor647, Cy5, and ATTO488 provided the most homogeneous rendered microtubule structures, followed by AlexaFluor568, Fluorescein, BODIPY FL, and ATTO680. Cy3B had the worst fluorophore homogeneity across the microtubule structure and the lack of Cy3B fluorophores rendered required the quadrupling of image contrast to aid in visualization of the displayed images (Fig. 1a,b). The range in SMLM image quality was expected as the chosen imaging conditions were not optimized for each fluorophore, but rather kept constant to enable differences in resulting image quality to be attributed to the photoswitching properties of the selected fluorophore. SMLM image quality using the selected fluorophores could be improved for future studies by tailoring the imaging buffer and acquisition settings for each fluorophore^12^,^32^.

SMLM image quality of the rendered microtubules resulting from each fluorophore was quantified by measuring the average width (Fig. 2a) and continuity (Fig. 2b). The width was measured as the full width at half maximum (FWHM) of the microtubule structure and the continuity was defined as the photons detected per nm^2^ of the measured microtubule. As expected, the average microtubule width measurements closely aligned with the observed quality of the rendered images (Figs 1a,b and 2a). The fluorophores with the narrowest average microtubule widths, AlexaFluor647 (51.3 ± 12.6 nm), Cy5 (60.2 ± 15.3 nm), and ATTO488 (62.9 ± 10.6 nm) had the highest quality microtubule images. These widths closely aligned with the expected 55 nm FWHM width, which are wider than the reported 25 nm width of microtubules as measured by electron crystallography due to the primary and secondary antibodies used for indirect immunofluorescence labeling^9^,^18^,^33^,^34^. Microtubule images of lower quality had larger widths including AlexaFluor568 (67.4 ± 20.7 nm), Fluorescein (76.1 ± 14.0 nm) and BODIPY FL (80.9 ± 23.1 nm). The poorest quality SRM images demonstrated the widest microtubules, which were found to be ATTO680 (83.1 ± 17.6 nm) and Cy3B (83.7 ± 37.3 nm) herein. The large microtubule width standard deviations reflect the inconsistent detection of fluorophores across even the most continuous microtubules within the SMLM image, which was likely attributed to their poor photoswitching properties and relatively low signal to background ratio (SBR). The average continuity measurements (Fig. 2b) also varied between fluorophores and showed a similar trend to observed image quality. Continuity was greatest for ATTO488 (1.48 ± 0.26 photons/nm^2^), AlexaFluor568 (1.35 ± 0.42 photons/nm^2^), Cy5 (1.20 ± 0.31 photons/nm^2^), and AlexaFluor647 (1.16 ± 0.29 photons/nm^2^). Fluorescein (1.11 ± 0.22 photons/nm^2^), ATTO680 (1.07 ± 0.23 photons/nm^2^), and BODIPY FL (0.97 ± 0.28 photons/nm^2^) demonstrated mid-level continuity, while Cy3B (0.52 ± 0.24 photons/nm^2^) showed the lowest average signal intensity and thus the least microtubule continuity. SMLM microtubule width and continuity measurements were used to identify correlation of image quality to photoswitching properties measured using each single-molecule isolation system.

### Correlation of SMLM Image Quality to Fluorophore Photoswitching Properties

**Figure 3:**
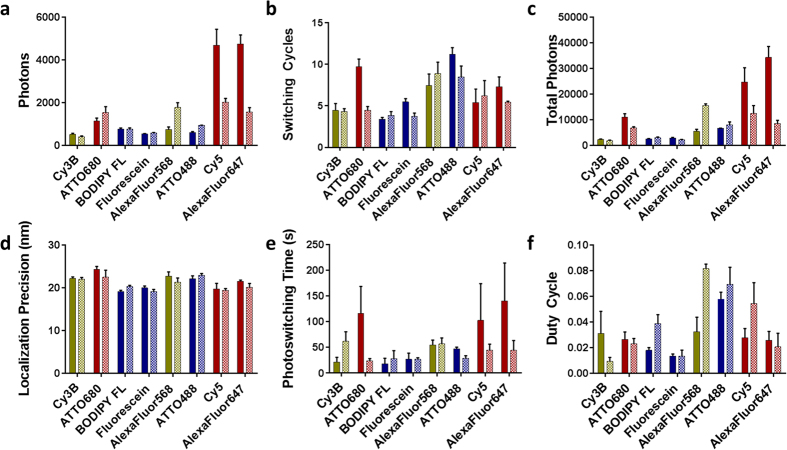
Photoswitching properties of antibody adsorption (solid bars) and PVA film (patterned bars) isolated fluorophores. Average photoswitching properties including (**a**) photons per switching cycle, (**b**) number of switching cycles, (**c**) total photons, (**d**) localization precision, (**e**) photoswitching time, and (**f**) duty cycle are demonstrated. Average photoswitching properties represented the mean ± standard deviation of n = 3 SMLM imaging series for each fluorophore. Photoswitching property measurements were collected at fluence rates equivalent to those utilized for imaging: 0.28 kWcm^−2^ for the 488 nm laser line (blue bars), 0.49 kWcm^−2^ for the 561 nm laser line (green bars) and 1.11 kWcm^−2^ for the 647 nm laser line (red bars).

The photoswitching properties of the eight fluorophores were measured using both the antibody adsorption and PVA film single-molecule isolation systems (Table 1 and Fig. 3a–f). Previous studies of a variety of commercially available photoswitchable fluorophores^35^,^36^,^37^,^38^,^39^,^40^ have shown that SMLM image quality is influenced by photoswitchable fluorophore photon output and the amount of time each fluorophore spends in the fluorescent on and off states^18^,^41^,^42^. Herein, we quantified six photoswitching properties for the spatially isolated fluorophores where four properties characterized photon output and two properties characterized time in the fluorescent on and off states. Measurements were collected in triplicate to assess photoswitching property reproducibility over three fluence rates within each excitation laser line. Measurements across the three fluence rates were used to assess the relationship between photoswitching behavior in each single-molecule isolation system and fluence rate. Measurements collected at the highest fluence rate were used for photoswitching property correlation analysis, as this was the same fluence rate used to acquire the SMLM microtubule images.

Of the six measured photoswitching properties, four characterized single-molecule photon output including: (1) photons per switching cycle, which represented the intensity of each molecule per fluorescent on cycle (Supplementary Fig. S1a), (2) number of switching cycles, which were the number of transitions from the dark off state to the fluorescent on state and were counted when photons were emitted above a set threshold (Supplementary Fig. S1b), (3) total photons, which were calculated as the photons per switching cycle multiplied by the number of switching cycles, and (4) localization precision, which reflected the deviation in the x and y position of the location of the maximum intensity of each molecule’s switching cycle^43^. The remaining two photoswitching properties characterized time in the fluorescent on and off states including: (5) photoswitching time, which reflected the length of time a molecule photoswitched before photobleaching occurred (Supplementary Fig. S1b), and (6) duty cycle, which described the fraction of time the molecule was on during the entire collected video.

Trends were seen when comparing the image quality of the eight selected fluorophores (Fig. 1) to their photoswitching property data (Fig. 3 and Table 1). Fluorophores with higher quality images tended to have higher photons per switching cycle, a greater number of switching cycles, higher total photons, and higher duty cycles. Statistically significant correlations were identified using Spearman two-tail correlation tests^44^ with significance reported as p < 0.05, which validated our observations. Both the antibody adsorption and PVA film systems produced total photon output results that correlated to SMLM image quality (Table 2) represented by the width (Antibody method: p = 0.037 and PVA method: p = 0.046). These findings agreed with previous studies that showed that SMLM image quality was improved by higher photon output as well as theoretical localization-precision-based calculations^16^,^18^,^19^. Additionally, both the antibody adsorption and PVA film systems produced duty cycle results that correlated to continuity measurements (Antibody method: p = 0.028 and PVA method: p = 0.037) (Table 2). Other photoswitching properties statistically correlated with continuity but the exact property varied based on the fluorophore isolation method used. Using the antibody adsorption method the localization precision correlated to continuity (p = 0.046). Using the PVA film isolation method, the switching cycles (p = 0.022) and total photons (p = 0.028) correlated to continuity (Table 2).

### Equivalent Fluorophore Photoswitching Property Measurements Using Antibody Adsorption and PVA Film Fixation Methods

The antibody adsorption fluorophore isolation method recapitulated established photoswitching property measurements collected using similar fixation techniques^10^,^18^. Comparing photons per switching cycle and number of switching cycles with the previously published literature demonstrated similar trends with our antibody isolated fluorophore photoswitching data. Both studies demonstrated similar photon output for the blue fluorophores ATTO488 and Fluorescein. When comparing photon output of the green fluorophores, AlexaFluor568 had higher photon output than Cy3B, similar to previously published results. Both studies also demonstrated a similar trend for the red fluorophores where AlexaFluor647 and Cy5 showed higher photon output than ATTO680^18^. Switching cycles were also comparable between the two studies, where ATTO488 had more switching cycles than Fluorescein for the blue fluorophores and Cy3B showed a similar number of switching cycles to AlexaFluor568 for the green fluorophores. The only anomaly between our photoswitching property data and the previously published results was in the switching cycle number for the red fluorophores. While both studies reported AlexaFluor647 had more switching cycles than Cy5, Dempsey *et al.* reported ATTO680 had the fewest switching cycles, while we measured the greatest number of switching cycles from ATTO680 across the red fluorophores^18^. Of note, the exact numerical values for photons per switching cycle and number of switching cycles between our study and previously published work did differ as would be expected from collecting photoswitching property data using different instrument configurations. The difference in switching cycle number across the red fluorophores was most likely due to differences in illumination fluence between the two studies, which strongly affect number of switching cycles. Even with some slight discrepancies, our antibody fluorophore isolation data demonstrated very similar photoswitching properties to previously published literature, validating our assay for photoswitching property evaluation. Of note, we did not directly compare duty cycle to the previously published study as our calculation represented an average over the entire collected dataset rather than a subset of frames that excluded the initial photobleach step. The inclusion of data collected during the initial photobleaching step in our duty cycle calculation resulted in higher absolute numerical values than previously reported^18^. Localization precision was also not directly compared to previous studies, as it has not previously been calculated for a suite of fluorophores.

The validated antibody adsorption photoswitching properties were compared to the PVA film isolated photoswitching properties to determine the similarity of the fluorophore photoswitching performance in these different environments. All six measured photoswitching properties showed similar overall trends across the eight fluorophores between the antibody adsorption and PVA film isolation methods at the high fluence rate (Table 1), which was used to collect the SMLM images utilized to assess image quality. Localization precision had the strongest trend with antibody adsorption values closely matching PVA film values for all eight fluorophores tested (Fig. 3d). Photons per switching cycle (Fig. 3a), number of switching cycles (Fig. 3b), and total photons (Fig. 3c) also showed strong trends between the antibody adsorption and PVA film photoswitching property data. Duty cycle (Fig. 3f) and photoswitching time (Fig. 3e) trended together less strongly than the other measured photoswitching properties between the two different fluorophore isolation methods. Overall, the antibody and PVA fluorophore isolation methods demonstrated strongly correlated photoswitching properties, which was further demonstrated by the fact that the total photons measured using each method statistically correlated to microtubule width and that the duty cycle measured for each method statistically correlated to microtubule continuity (Table 2).

**Figure 4:**
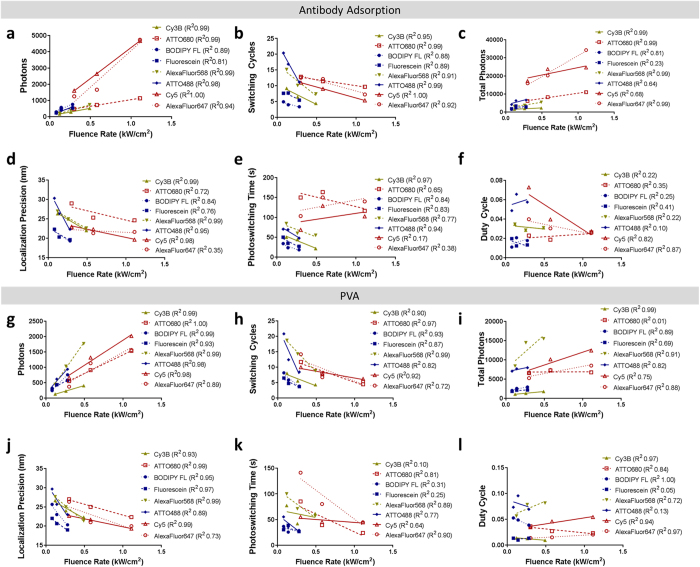
Correlation of photoswitching properties and fluence rate for antibody adsorption (**a**–**f**) and PVA (**g**–**l**) isolated fluorophores. Linear regression analysis of each average photoswitching property versus fluence rate was completed and R^2^ values were calculated (see key for values) for (**a,g**) photons per switching cycle, (**b,h**) switching cycles, (**c,i**) total photons, (**d,j**) localization precision, (**e,k**) photoswitching time, and (**f,l**) duty cycle. Mean photoswitching properties for n = 3 SMLM imaging series for each fluorophore are displayed.

Correlations seen between the photoswitching properties measured with both the antibody adsorption and PVA film isolation methods at the highest fluence rate translated to trends seen across all three fluence rates (Fig. 4). Localization precision (Fig. 4d,j) and switching events (Fig. 4b,h) demonstrated the strongest correlation at the highest fluence rate. Photons (Fig. 4a,g) and total photons (Fig. 4c,i) trended similarly for both the antibody adsorption and PVA film isolation methods. Photoswitching time (Fig. 4e,k) and duty cycle (Fig. 4f,l) showed little relationship to fluence rate.

### Effect of Excitation Fluence Rate on Photoswitching Properties

Correlative relationships were observed between select photoswitching properties and fluence rate using both the antibody adsorption and PVA film single-molecule systems (Fig. 4). Linear regressions were calculated using the average value (n = 3 SMLM image series/fluorophore) for each photoswitching property at each fluence rate to quantify the degree of correlation using R^2^ values. Photons per switching cycle (Antibody R^2^ = 0.81–1.0, PVA R^2^ = 0.89–1.0) and number of switching cycles (Antibody R^2^ = 0.88–1.0, PVA R^2^ = 0.72–0.99) were strongly correlated with fluence rate, where increased fluence rate resulted in greater photon output and fewer switching cycles. Total photons (Antibody R^2^ = 0.23–0.99, PVA R^2^ = 0.01–0.99) and localization precision (Antibody R^2^ = 0.35–0.99, PVA R^2^ = 0.73–0.99) showed general correlation with fluence rate, however neither property was as strongly correlated as photons per switching cycle or number of switching cycles. Photoswitching time and duty cycle were largely unaffected by fluence rate (Fig. 4e,f,k,l).

### Intersample Stability of Photoswitching Property Measurements

The reported photoswitching properties represented mean and standard deviation calculated from triplicate imaging series collected for each fluorophore at three fluence rates (Table 1 and Supplementary Figs S2 and S3). The coefficient of variation (CV) was less than 1 for all fluorophores across the computed photoswitching properties, demonstrating that the photoswitching property measurements were reproducible using both the antibody adsorption and PVA film fluorophore isolation methods. Some properties had lower standard deviation than others exhibiting better reproducibility. For both the antibody adsorption and PVA film fixation methods the least variable photoswitching property was localization precision (CV < 0.1 for all fluorophores), followed by photons with CVs ranging 0.03–0.4. The most variable photoswitching properties was photoswitching time with CVs ranging up to 0.7. However, overall both fluorophore isolation methods provided robust, reproducible measurements of the defined photoswitching properties.

## Discussion

In summary, we developed a methodology to correlate fluorophore photoswitching properties to SMLM image quality using two single molecule isolation systems. We examined PVA film as a method for facile characterization of fluorophores developed for SMLM or optimization of imaging buffer systems, where the photoswitching properties of fluorophores measured using both PVA film and the previously utilized antibody adsorption method were correlated with SMLM image quality (Figs 1 and 2). While prior studies have characterized photoswitching^10^,^14^,^18^, our study is the first to demonstrate a statistically significant correlation between SMLM image quality and photoswitching properties including total photons and duty cycle (Table 2). We found microtubule width was statistically correlated with total photon output using both the antibody absorption and PVA film fluorophore isolation methods, while duty cycle was significantly correlated to microtubule continuity using both fluorophore isolation methods (Table 2 and Fig. 3). Interestingly, previous studies have demonstrated strong correlation between photons per switching cycle and SRM image quality. In the current study, total photons was significantly correlated to image quality rather than photons per switching cycle, which is likely explained by the different photophysical environments provided by the antibody adsorption and PVA film isolation methods. Although the imaging buffer utilized in both fluorophore isolation systems contained oxygen scavengers, the PVA film may have further reduced oxygen permeation into the system, thus affecting photoswitching physics^6^,^22^,^45^, potentially accounting for the differences seen in photon output per switching cycle and the number of switching cycles compared with the antibody adsorption method. However, the calculation of total photon output as the product of these two photoswitching properties recapitulated the relationship seen in the antibody adsorption fluorophore isolation method, where significant correlation was demonstrated between image quality and overall photon output (Table 2).

Interestingly, we found that fluorophores resulting in higher quality images had higher duty cycle than those of lower quality SRM images, which is in contradiction to previous studies where shorter duty cycle was thought to improve SRM image quality^18^. We hypothesize that higher duty cycle was significantly correlated with image quality in our study because of the difference in duty cycle calculation. In our study, duty cycle was calculated over the entire collected photoswitching analysis video, where images were collected as soon as the laser was turned on and thus, no pre-photobleaching step was included. Therefore, our higher duty cycle measurements represent individual fluorophore molecules that were less susceptible to photobleaching. Thus, higher duty cycles reflected fluorophore molecules spending more time in the fluorescent on state. While higher duty cycles could enhance imaging by improving the ability to detect individual fluorophore molecules, it could potentially be detrimental for fluorophores to have high cycles as it increases the chances of detected fluorophore overlap, which degrades SMLM image quality^46^. The low fluorophore density used for photoswitching property analysis in this study were well below the threshold where fluorophore overlap would affect accurate quantification of photoswitching property measurements, explaining the strong imaging quality correlation to the higher duty cycle photoswitching property in both the antibody absorption and PVA fluorophore isolation methods.

We also found that the photon output related photoswitching properties measured using both fluorophore isolation methods correlated to fluence rate, while the fluorescent on and off time parameters showed little correlation with fluence rate. While previous studies observed that the fluence rate used to excite photoswitchable fluorophores affects SMLM image quality^18^,^47^, the clear impact of fluence rate on photoswitching properties and image quality was demonstrated here. We found a strong correlation between photons per switching cycle and number of switching cycles as well as general correlation between fluence rate, total photons and localization precision. Higher quality SMLM images were achieved with fluorophores having higher total photons and duty cycle (Figs 1 and 3). Therefore, the highest fluence rate tested for each laser line was found to be the best for SMLM imaging, and hence most effective for future screening of developed fluorophores for SMLM. Importantly, similarities in photoswitching property rankings and response to fluence rate will enable either fluorophore isolation method to be used for accurate fluorophore evaluation and comparison. Lastly, we also quantified the repeatability of photoswitching measurements in both the antibody adsorption and PVA film based systems, demonstrating a robust platform for screening developed fluorophores for SMLM using single-molecule isolation (Fig. 3 and Table 1).

Herein, we demonstrate that PVA is a facile fluorophore isolation method that can be used to screen fluorophores or imaging buffer conditions to predict SMLM image quality based on the quantification of total photon output and duty cycle. The PVA film method eliminates the functionality hurdle in screening novel fluorophores, facilitating a potentially high throughput approach for large studies. The utilization of PVA film fluorophore isolation system provides the necessary means to evaluate and guide future fluorophore development, as well as screen for optimal image buffer conditions, to improve the spatial and spectral resolution of SRM through quantification of photoswitching properties that predict SRM image quality.

## Methods

### Fluorophores

The eight fluorophores utilized in this study were obtained commercially in their succinimidyl ester form including Fluorescein, BODIPY FL, AlexaFluor568, AlexaFluor647 (Thermo Fisher Scientific), ATTO488, ATTO680 (ATTO-TEC), Cy3B and Cy5 (GE Healthcare Life Sciences).

### Fluorophore-labeled Antibodies

Each fluorophore was conjugated to donkey anti-mouse antibody (Jackson ImmunoResearch) for SMLM microtubule imaging and single-molecule photoswitching property measurements. The antibody was buffer exchanged into 1x phosphate buffered saline (PBS) and pH adjusted to 8.0 with 35 mM disodium phosphate. The conjugation reactions were set up with fluorophore and antibody mixed at a 5:1 molar ratio for SMLM microtubule imaging and 1:1 molar ratio for single-molecule photoswitching property measurements. The fluorophore-antibody conjugation reactions were rocked gently at room temperature protected from light for 3 hr and were concentrated in 10 kDa MWCO spin filters followed by purification using a 7 kDa desalting column. The fluorophore to antibody-labeling conjugation ratios ranged from 1.1:1 to 2.3:1 for SMLM microtubule imaging and ranged from 0.4:1 to 0.5:1 for single-molecule photoswitching property measurements, which were determined using an absorbance spectrometer (SpectraMax M5 Microplate Reader).

### Single-Molecule Localization Super Resolution Microscope Configuration

Imaging was completed on a Nikon ECLIPSE Ti-U inverted microscope equipped with a 60x oil immersion objective (NA = 1.49) using total internal reflection fluorescence configuration of the light path. Excitation laser lines included 488-nm (Coherent), 561-nm (Opto Engine LLC), and 647-nm (Coherent), with images collected through a 525/45, 605/64, or 708/75 nm single-bandpass filter (Semrock Inc.), respectively. An EMCCD camera (Andor Technology) recorded images in a 512 × 512 pixel area at 107 nm/pixel, with a 100 ms exposure time and a EM gain setting of 300 via Micro-Manager^48^,^49^. Images of single molecules were processed with modified custom-written Matlab scripts (Mathworks)^50^. Microtubule images were processed with the ThunderSTORM^51^ plugin for ImageJ where the approximate localization of individual fluorophores was identified as signal intensities above a set threshold. Image processing conditions were kept constant across all 8 selected fluorophores.

### Imaging Buffer

Tris-buffered saline (TN buffer, 50 mM Tris pH 8.0 and 10 mM NaCl) with oxygen scavenger components including 0.5 mgml^−1^ glucose oxidase, 40 μgml^−1^ catalase (Sigma Aldrich), and 10% w/v glucose, as well as the reducing component 10 mM β-mercaptoethylamine, were utilized for all imaging and photoswitching property measurements^18^,^52^.

### SMLM Microtubule Images

#### Cell Culture

U2OS cells were cultured in Dulbecco’s Modified Eagle media without phenol red supplemented with 10% FBS and 1% Penicillin-Streptomycin-Glutamine at 37 °C and 5% CO_2_. LabTek 8-well coverglass chambers were washed with SparKLEEN (5 min), milli-Q water (3 × 5 min), 1 M NaOH (90 min), and milli-Q water (3 × 5 min) prior to plating cells at ~80,000 cells per well. Cells were incubated for 2 days to reach ~75% confluency.

#### Immunofluorescence Labeling

Cells were preextracted with 0.5% Triton X-100 in PEM (100 mM PIPES buffer pH 7.0, 1 mM EGTA and 1 mM MgCl_2_) for 20 s, fixed with 0.4% glutaraldehyde (Electron Microscopy Science) and 0.25% Triton X-100 in PEM for 90 s, washed with PBS before fixing with 3% glutaraldehyde in PEM 15 min. Cells were washed with PBS (3 × 5 min), then reduced with 10 mM sodium borohydride for 10 min, washed again with PBS (3 × 5 min), and blocked with 5% bovine serum albumin (BSA) in PBS. Cells were incubated with bovine alpha-tubulin mouse primary antibody (Thermo Fisher Scientific) at 2 μgml^−1^ in BSA for 4 h at room temperature and washed with PBS (3 × 5 min). Lastly, cells were incubated with fluorophore-labeled donkey anti-mouse secondary antibody at 0.2 μM fluorophore concentration across the eight fluorophores in 5% BSA for 30 min at room temperature protected from light and washed with PBS (3 × 5 mins)^20^.

#### Imaging

A mixture of 200 μl imaging buffer and 2 μl gold nanoparticles (BBI International) fiducial markers was added to the labeled cells prior to imaging. Images were collected at a frame rate of 10 Hz for 10,000 frames. Samples labeled with Fluorescein, ATTO488, or BODIPY FL were excited using 488-nm laser line (0.28 kWcm^−2^). Samples labeled with Cy3B or AlexaFluor568 were excited by the 561-nm laser line (0.49 kWcm^−2^). Samples labeled with Cy5, AlexaFluor647, or ATTO680 were excited by the 647-nm laser line (1.11 kWcm^−2^). Imaging began with exposure to the excitation laser and a rapid decrease in the density of “on” fluorophores was observed within the first few hundred frames of the 10,000 frame movies, after which the observed “on” fluorophores in each frame were spatially separated.

#### Microtubule Quantitation

The image quality of the rendered SMLM images was quantitated based on the microtubule width and continuity, which were measured at 15 representative points per image where labeling was most complete and the structure was well defined. The width was calculated by taking the FWHM from line profiles summed over 13 nm lengths of the microtubule structure. The continuity was reported as the photon density of microtubules per square nanometer. Continuity was calculated as the summed photons detected along a 670 nm length sections of microtubules using the calculated width to determine the measured microtubule area in nm^2^.

### Single-molecule Fixation

Single-molecule samples were fixed in 96-well glass bottom plates with #1.5 coverglass bottom (*In Vitro* Scientific) and washed as detailed for the LabTek plates above. Single-molecule isolation was performed using two fixation methods: antibody adsorption and PVA film formation. For the antibody adsorption method, 100 μl of fluorophore-labeled antibody diluted to a final concentration of 1 × 10^−10^ M fluorophore in 1x PBS was used per well. Antibody solutions were incubated for 16 h at room temperature sealed and protected from light, then washed and stored in PBS at 4 °C. For the PVA film method, fluorophores were diluted to a final concentration of 5 × 10^−9^ M in 1 wt% PVA (72,000 MW) and 50 μl was used per well before drying in a fume hood overnight. Sample preparation resulted in 150–300 observed particles dispersed throughout the 512 × 512 pixel field of view. At 107 nm/pixel, this equated to a density of 0.05–0.1 fluorophores/μm^2^.

### Single-molecule Photoswitching Measurements

All measurements were carried out in the imaging buffer described above. For adsorbed antibody samples, storage PBS was aspirated prior to the addition of imaging buffer. For PVA film samples, the dried PVA films were quickly flushed with PBS 3x to remove any molecules not isolated by the dried film prior to addition of imaging buffer. PVA film measurements were collected between 10 and 60 minutes after adding the imaging buffer to allow for adequate penetration of the buffer into the solid film, but prior to any compromise of the film by rehydration that could allow for molecule drift out of the PVA matrix.

Each sample was imaged three times in three different regions of the 96-well plate at each of the selected fluence rates termed low, mid and high for each laser line. Fluorescein, ATTO488 and BODIPY FL were excited with a 488-nm laser line at fluence rates of 0.08, 0.14, and 0.28 kWcm^−2^. Cy3B and AlexaFluor568 were excited with a 561-nm laser line at fluence rates of 0.12, 0.26, and 0.49 kWcm^−2^. Cy5, AlexaFluor647, and ATTO680 were excited with a 647-nm laser line at fluence rates of 0.30, 0.58, and 1.11 kWcm^−2^. Imaging videos were collected for 5,000 frames at 10 Hz.

### Single-molecule Data Analysis

Single molecules were identified as fluorescent signals detected above a threshold 6 times root mean square (RMS) of the average detected signal. Molecules were tracked positionally throughout the 5,000-frame image series while recording the x and y coordinates of their point-spread function. A minimum of 100 molecules were identified in each 5,000-frame image series. Photoswitching properties were quantitated as follows.

#### Photons per Switching Cycle

The number of photons emitted per switching cycle was calculated algebraically by summing the fluorescence emission signal collected over consecutive frames from a single molecule that was above 6 times RMS, minus the baseline intensity. This was considered a single switching cycle. The summed signal (analog digital units, ADU) was converted to photons using the camera’s reported analog-to-digital conversion factor (DCF, electrons/ADU), quantum efficiency (QE), and the acquisition gain setting (gain) as follows: photons = ADU x DCF x 1/QE x 1/gain.

#### Switching Cycles

The number of switching cycles was determined by counting the instances a tracked particle emitted photons above 6 times RMS, reflecting how often the molecule transitioned from the off to the on state.

#### Total Photons

The total photon values were calculated by multiplying the photons per switching cycle by the number of switching cycle, resulting in the total photons a single molecule emitted throughout the image series.

#### Localization Precision

The lateral localization precision, σ_xy_, was determined using the standard deviation of the x (σ_x_) and y (σ_y_) coordinates recorded for each molecule throughout the 5,000-frame image series calculated as σ_xy_ = (σ_x_^2^ + σ_y_^2^)^1/2^.

#### Photoswitching Time

The photoswitching time was calculated as the amount of time in seconds a single molecule photoswitched during the entire 5,000-frame image series. This was calculated as the time difference between the first switching cycle and the last switching cycle. Results were calculated only for molecules that had their initial cycle during the first 100 s to prevent truncating photoswitching time calculations for molecules that started blinking at a later time point in the image series.

#### Duty Cycle

The duty cycle represents the fraction of time fluorophores emit photons during the 500 s acquisition time. Duty cycle was calculated as the number of frames the fluorophore was considered on (above the set threshold of RMS 6) divided by the total number of frames.

### Statistical Analysis of Photoswitching Properties

Statistical analysis was completed using GraphPad Prism (GraphPad Software). To determine the correlation of each photoswitching property to image quality, a spearman two-tail correlation test^44^ was completed comparing the set of average widths (n = 15/fluorophore, 8 fluorophores) and the set of average continuity (n = 15/fluorophore, 8 fluorophores) to the average of each photoswitching property (n = 3/fluorophore, 8 fluorophores) (Table 2). This test was conducted on data from both the antibody adsorption and PVA film fixation methods. Correlation was reported as significant for p < 0.05.

To study the relationship between fluence rate and photoswitching properties, linear regressions were completed using the average value (n = 3 SMLM image series/fluorophore) for a particular photoswitching property at each fluence rate. Computed R^2^ values, defined as the coefficient of determination, were reported to characterize the correlation between photoswitching property and fluence rate. R^2^ values closer to 1 indicate a more linear relationship while R^2^ values closer to 0 indicate a less linear relationship^53^.

To study the intersample stability of photoswitching property measurements, the coefficient of variance (CV) was calculated by dividing the standard deviation of the triplicate measurements by the mean of the triplicate measurements.

## Additional Information

**How to cite this article**: Bittel, A. M. *et al.* Methodology for Quantitative Characterization of Fluorophore Photoswitching to Predict Superresolution Microscopy Image Quality. *Sci. Rep.* **6**, 29687; doi: 10.1038/srep29687 (2016).

## Supplementary Material

Supplementary Information

## Figures and Tables

**Table 1:** Photoswitching properties of 8 fluorophores isolated via antibody adsorption and PVA methods.

	Fluorophore		Photons	Switching Cycles	Total Photons	Localization Precision (nm)	Photoswitching Time (s)	Duty Cycle
Antibody Adsorption	Cy3B	low Φ	169 ± 26	9.2 ± 1.6	1,535 ± 116	26.6 ± 0.8	50 ± 2	0.0348 ± 0.0099
		mid Φ	288 ± 12	6.5 ± 0.9	1,871 ± 190	25.1 ± 0.1	45 ± 17	0.0286 ± 0.0048
		high Φ	515 ± 50	4.5 ± 0.8	2,265 ± 229	22.2 ± 0.4	21 ± 9	0.0311 ± 0.0172
	ATTO680	low Φ	477 ± 34	12.9 ± 0.6	6,143 ± 721	28.7 ± 1.9	150 ± 51	0.0230 ± 0.0049
		mid Φ	721 ± 60	11.4 ± 0.9	8,283 ± 1,278	25.6 ± 0.6	164 ± 31	0.0187 ± 0.0017
		high Φ	1136 ± 139	9.7 ± 0.9	11,002 ± 1,362	24.4 ± 0.7	116 ± 52	0.0263 ± 0.0059
	BODIPY FL	low Φ	294 ± 13	4.9 ± 0.3	1,456 ± 140	22.7 ± 0.6	34 ± 14	0.0111 ± 0.0021
		mid Φ	570 ± 198	4.0 ± 0.6	2,221 ± 520	20.3 ± 1.1	23 ± 7	0.0207 ± 0.0079
		high Φ	756 ± 57	3.4 ± 0.2	2,555 ± 106	19.2 ± 0.3	18 ± 11	0.0179 ± 0.0021
	Fluorescein	low Φ	240 ± 16	7.6 ± 0.3	1,837 ± 184	22.1 ± 0.6	50 ± 8	0.0198 ± 0.0041
		mid Φ	444 ± 32	7.7 ± 1.0	3,415 ± 197	20.5 ± 0.7	35 ± 7	0.0123 ± 0.0039
		high Φ	531 ± 21	5.5 ± 0.4	2,908 ± 207	20.0 ± 0.5	27 ± 12	0.0133 ± 0.0017
	AlexaFluor568	low Φ	206 ± 34	15.2 ± 1.9	3,126 ± 670	27.0 ± 0.6	85 ± 18	0.0338 ± 0.0008
		mid Φ	389 ± 97	10.1 ± 2.4	3,796 ± 368	25.0 ± 0.3	60 ± 12	0.0324 ± 0.0027
		high Φ	739 ± 124	7.5 ± 1.4	5,438 ± 854	22.7 ± 1.0	55 ± 9	0.0324 ± 0.0113
	ATTO488	low Φ	205 ± 12	20.4 ± 4.3	4,158 ± 763	30.4 ± 1.1	70 ± 14	0.0487 ± 0.0088
		mid Φ	374 ± 39	16.9 ± 2.4	6,362 ± 1,542	26.0 ± 0.6	69 ± 20	0.0657 ± 0.0065
		high Φ	595 ± 53	11.2 ± 0.8	6,638 ± 97	22.2 ± 0.7	47 ± 3	0.0576 ± 0.0056
	Cy5	low Φ	1,606 ± 675	11.0 ± 2.5	17,174 ± 7,080	23.0 ± 0.9	68 ± 29	0.0728 ± 0.0091
		mid Φ	2,643 ± 115	9.0 ± 1.7	23,808 ± 3,788	22.2 ± 0.6	129 ± 36	0.0396 ± 0.0058
		high Φ	4682 ± 749	5.4 ± 1.6	24,635 ± 5,635	19.8 ± 1.3	103 ± 71	0.0275 ± 0.0074
	AlexaFluor647	low Φ	1,255 ± 115	12.6 ± 1.9	15,913 ± 3,740	22.7 ± 1.4	103 ± 48	0.0399 ± 0.0064
		mid Φ	1,663 ± 74	12.2 ± 1.5	20,205 ± 1,827	21.5 ± 1.0	151 ± 71	0.0304 ± 0.0030
		high Φ	4742 ± 427	7.3 ± 1.2	34,309 ± 4,253	21.5 ± 0.3	140 ± 74	0.0257 ± 0.0069
**PVA**	Cy3B	low Φ	133 ± 4	8.0 ± 1.8	1,065 ± 225	26.5 ± 1.4	77 ± 15	0.0138 ± 0.0058
		mid Φ	230 ± 27	5.6 ± 0.8	1,269 ± 181	23.8 ± 0.9	42 ± 16	0.0127 ± 0.0026
		high Φ	405 ± 40	4.3 ± 0.3	1,756 ± 293	22.0 ± 0.4	62 ± 19	0.0093 ± 0.0031
	ATTO680	low Φ	555 ± 106	11.7 ± 2.4	6,576 ± 2,096	26.7 ± 1.1	85 ± 49	0.0364 ± 0.0101
		mid Φ	912 ± 103	8.0 ± 1.3	7,249 ± 1,002	24.9 ± 0.8	39 ± 7	0.0270 ± 0.0071
		high Φ	1,536 ± 275	4.5 ± 0.5	6,762 ± 526	22.5 ± 1.6	24 ± 4	0.0231 ± 0.0040
	BODIPY FL	low Φ	247 ± 9	8.2 ± 0.8	2,020 ± 208	25.9 ± 1.5	36 ± 15	0.0525 ± 0.0101
		mid Φ	434 ± 21	5.9 ± 0.4	2,561 ± 200	23.0 ± 0.4	26 ± 10	0.0486 ± 0.0176
		high Φ	758 ± 60	3.9 ± 0.4	2,926 ± 361	20.3 ± 0.3	28 ± 15	0.0387 ± 0.0070
	Fluorescein	low Φ	277 ± 7	6.5 ± 0.3	1,797 ± 71	21.8 ± 0.6	30 ± 11	0.0134 ± 0.0027
		mid Φ	439 ± 29	4.7 ± 0.6	2,086 ± 340	20.3 ± 0.7	39 ± 21	0.0099 ± 0.0046
		high Φ	579 ± 23	3.7 ± 0.4	2,146 ± 158	19.1 ± 0.5	27 ± 3	0.0134 ± 0.0046
	AlexaFluor568	low Φ	447 ± 66	18.7 ± 1.7	8,392 ± 1,793	27.3 ± 0.3	100 ± 4	0.0563 ± 0.0070
		mid Φ	1,309 ± 232	14.3 ± 2.5	14,482 ± 788	25.1 ± 0.2	71 ± 16	0.0785 ± 0.0170
		high Φ	1,772 ± 227	8.9 ± 1.4	15,506 ± 699	21.3 ± 1.0	57 ± 11	0.0815 ± 0.0036
	ATTO488	low Φ	340 ± 22	20.8 ± 3.8	7,053 ± 1,106	29.9 ± 2.5	55 ± 11	0.0734 ± 0.0161
		mid Φ	619 ± 35	12.4 ± 0.5	7,681 ± 255	25.6 ± 0.2	36 ± 0	0.0955 ± 0.0099
		high Φ	940 ± 7	8.5 ± 1.3	7,962 ± 1,177	22.9 ± 0.4	29 ± 5	0.0693 ± 0.0133
	Cy5	low Φ	670 ± 146	10.0 ± 2.4	6,711 ± 2,334	22.7 ± 0.6	55 ± 8	0.0346 ± 0.0074
		mid Φ	1,317 ± 204	7.7 ± 0.6	10,125 ± 1,048	21.4 ± 1.1	45 ± 14	0.0459 ± 0.0083
		high Φ	2,021 ± 175	6.2 ± 1.8	12,417 ± 3,118	19.5 ± 0.4	45 ± 11	0.0543 ± 0.0163
	AlexaFluor647	low Φ	374 ± 37	14.2 ± 2.4	5,248 ± 445	26.2 ± 1.8	141 ± 17	0.0138 ± 0.0022
		mid Φ	1,137 ± 261	6.8 ± 1.9	7,377 ± 1,006	21.0 ± 1.1	80 ± 21	0.0151 ± 0.0019
		high Φ	1,566 ± 201	5.4 ± 0.1	8,506 ± 1,270	20.2 ± 0.9	45 ± 19	0.0205 ± 0.0107

Results are mean ± standard deviation; N = 3 SMLM movies for each fluorophore, with at least 100 particles analyzed in each movie. Fluence rate is represented by Φ. BODIPY FL, Fluorescein, and ATTO488 and were excited with the 488 nm laser line, with low Φ = 0.08, mid Φ = 0.14, and high Φ = 0.28 kW cm^−2^. Cy3B and AlexaFluor568 were excited with the 561 nm laser line, with low Φ = 0.12, mid Φ = 0.26, and high Φ = 0.49 kW cm^−2^. ATTO680, Cy5, and AlexaFluor647 were excited with the 647 nm laser line, with low Φ = 0.30, mid Φ = 0.58, and high Φ = 1.11 kW cm^−2^.

**Table 2:** P value results: photoswitching properties correlated to image quality via width and continuity for antibody adsorption and PVA single-molecule systems.

Photoswitching Property	Width		Continuity	
	Antibody	PVA	Antibody	PVA
Photons	0.132	0.069	0.501	0.096
Switching Cycles	0.501	0.171	0.327	0.022*
Total Photons	0.037*	0.046*	0.882	0.028*
Localization Precision	0.428	0.389	0.046*	0.882
Photoswitching Time	0.151	0.752	0.840	0.793
Duty Cycle	0.840	0.299	0.028*	0.037*

P values calculated via two-tailed Spearman correlation, with *p < 0.05.
