# Supplementary material for: Methodology for Quantitative Characterization of Fluorophore Photoswitching to Predict Superresolution Microscopy Image Quality
Source: Sci Rep. 2016 Jul 14;6:29687. doi: 10.1038/srep29687 (PMC4944197; doi:10.1038/srep29687)
Supplement: Supplementary Information [file srep29687-s1.pdf]

Supporting Information

Methodology for Quantitative Characterization of Fluorophore Photoswitching to Predict Superresolution Microscopy Image Quality

Amy M. Bittel[1], Andrew Nickerson[1], Isaac S. Saldivar[1], Nick J. Dolman[4], Xiaolin Nan[1,2,3], Summer L. Gibbs[1,2,3]

Biomedical Engineering Department[1], Knight Cancer Institute[2], OHSU Center for Spatial Systems Biomedicine[3], Oregon Health & Science University, Portland, OR 97201

Thermo Fisher Scientific, Eugene, OR 97402[4]

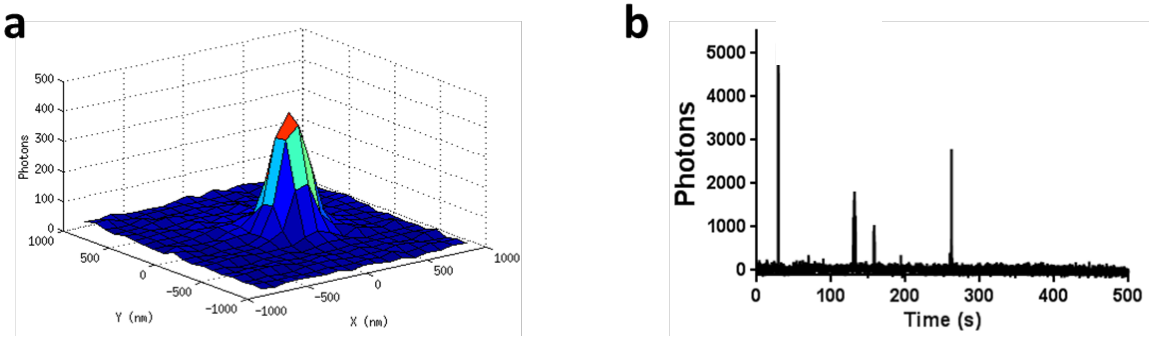

Figure S1. Representative single molecule data to demonstrate photoswitching property calculations. Photoswitching data characterizing a single molecule of AlexaFluor568 fixed in PVA film, measured at the high fluence rate ($\Phi = 0.49$ kWcm^{-2}) are shown. (a) The intensity of a single molecule in its fluorescent on state in one frame. (b) Intensity tracked throughout the entire 500 s imaging series. Switching events represented the number of spikes in intensity over a set threshold. Photoswitching time represented the time from the first switching event to the last switching event.

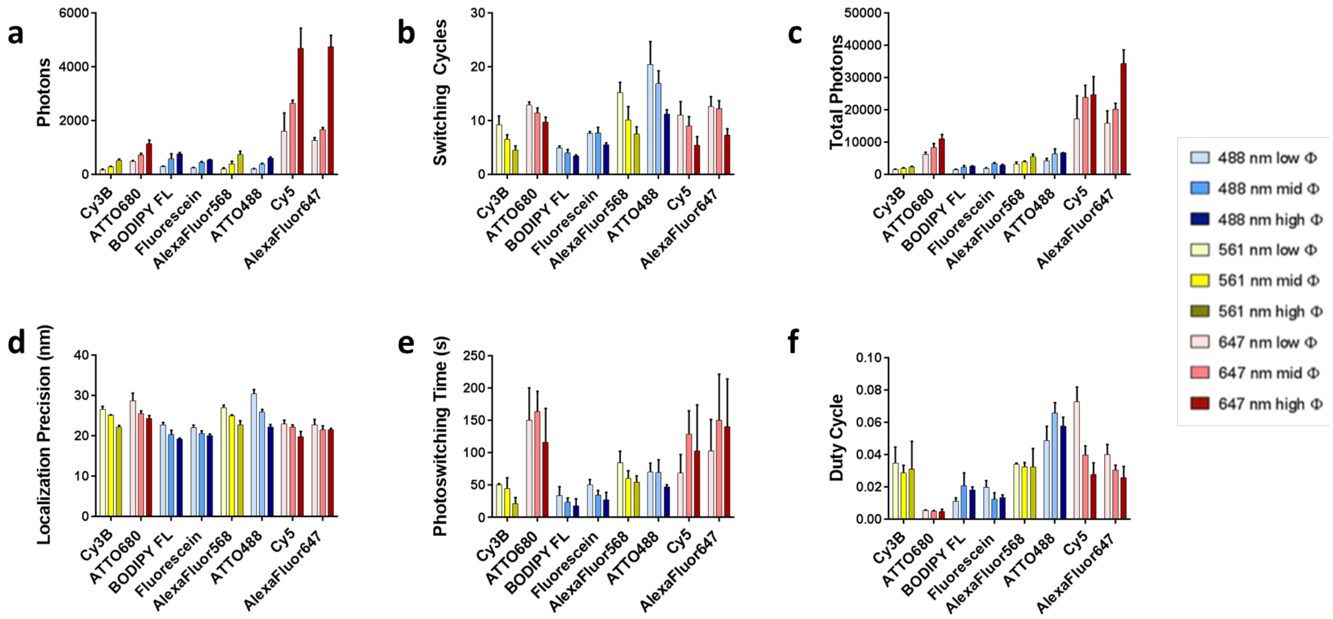

Figure S2. Photoswitching properties of antibody adsorption isolated fluorophores over a range of fluence rates. A low, mid and high fluence rate (Φ) were selected for each laser line and utilized to measure photoswitching properties including (a) photons per switching cycle, (b) number of switching cycles, (c) total photons, (d) localization precision, (e) photoswitching time, and (f) duty cycle. Average photoswitching properties represent the mean ± standard deviation of n=3 single molecule localization SRM imaging series for each fluorophore.

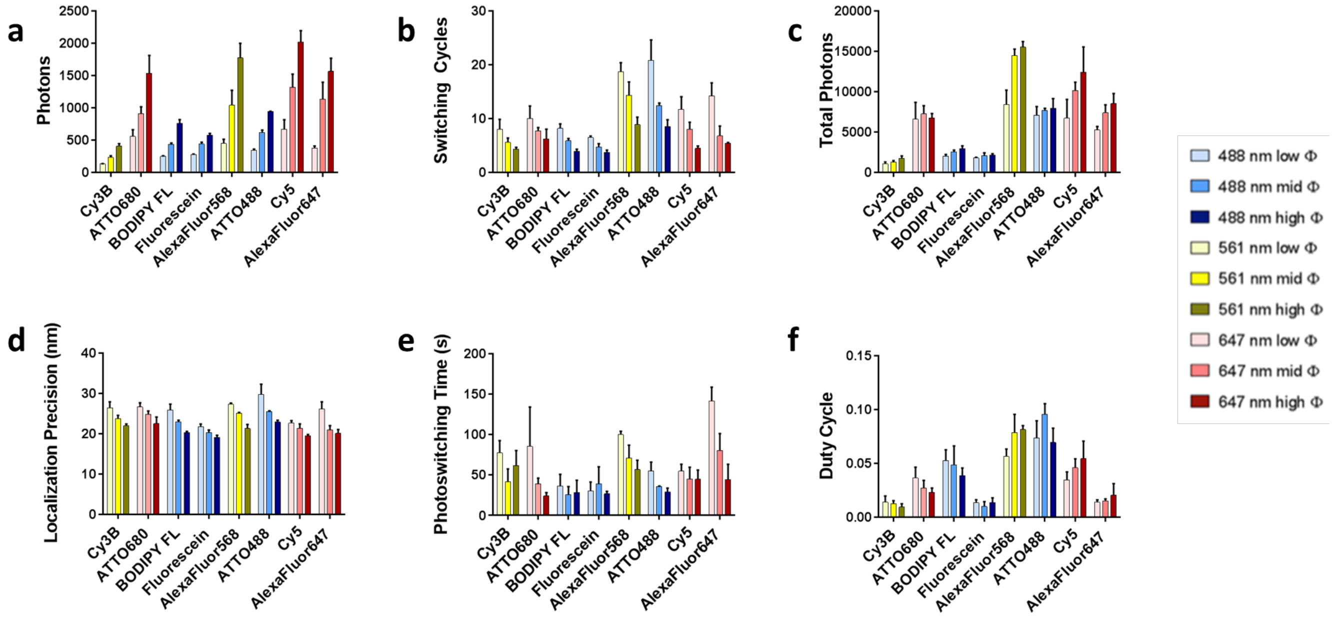

Figure S3. Photoswitching properties of PVA film isolated fluorophores over a range of fluence rates. A low, mid and high fluence rate (Φ) were selected for each laser line and utilized to measure photoswitching properties including (a) photons per switching cycle, (b) number of switching cycles, (c) total photons, (d) localization precision, (e) photoswitching time, and (f) duty cycle. Average photoswitching properties represent the mean ± standard deviation of n=3 single molecule localization SRM imaging series for each fluorophore.
